# ^13^C-sucrose breath test for the non-invasive assessment of environmental enteropathy in Zambian adults

**DOI:** 10.3389/fmed.2022.904339

**Published:** 2022-07-29

**Authors:** Robert J. Schillinger, Simutanyi Mwakamui, Chola Mulenga, Mizinga Tembo, Phoebe Hodges, Ellen Besa, Kanta Chandwe, Victor O. Owino, Christine A. Edwards, Paul Kelly, Douglas J. Morrison

**Affiliations:** ^1^Scottish Universities Environmental Research Centre, University of Glasgow, East Kilbride, United Kingdom; ^2^School of Medicine, Dentistry and Nursing, University of Glasgow, Glasgow, United Kingdom; ^3^Tropical Gastroenterology and Nutrition Group, University of Zambia School of Medicine, Lusaka, Zambia; ^4^Blizard Institute, Barts & The London School of Medicine, Queen Mary University of London, London, United Kingdom; ^5^Nutritional and Health-Related Environmental Studies Section, Division of Human Health, International Atomic Energy Agency (IAEA), Vienna, Austria

**Keywords:** environmental enteropathy, malnutrition, stunting, ^13^C-breath test, gut function, intestinal sucrase activity

## Abstract

**Objectives:**

Environmental enteropathy (EE) is a subclinical disorder highly prevalent in tropical and disadvantaged populations and is thought to play a role in growth faltering in children, poor responses to oral vaccines, and micronutrient deficiencies. This study aims to evaluate the potential of a non-invasive breath test based on stable isotopes for evaluation of impaired digestion and absorption of sucrose in EE.

**Methods:**

We optimized a ^13^C-sucrose breath test (^13^C-SBT) in 19 young adults in Glasgow, United Kingdom. In a further experiment (in 18 adults) we validated the ^13^C-SBT using Reducose, an intestinal glucosidase inhibitor. We then compared the ^13^C-SBT to intestinal mucosal morphometry, immunostaining for sucrose-isomaltase (SI) expression, and SI activity in 24 Zambian adults with EE.

**Results:**

Fully labeled sucrose (0.3 mg/kg) provided clear breath enrichment signals over 2–3 h in both British and Zambian adults, more than fivefold higher than naturally enriched sucrose. Reducose dramatically impaired ^13^C-sucrose digestion, reducing 4 h ^13^CO_2_ breath recovery by > 50%. Duodenal biopsies in Zambian adults confirmed the presence of EE, and SI immunostaining was present in 16/24 adults. The kinetics of ^13^CO_2_ evolution were consistently faster in participants with detectable SI immunostaining. Although sucrase activity was strongly correlated with villus height (*r* = 0.72; *P* < 0.05) after adjustment for age, sex and body mass index, there were no correlations between ^13^C-SBT and villus height or measured sucrase activity in pinch biopsies.

**Conclusion:**

A ^13^C-SBT was developed which was easy to perform, generated clear enrichment of ^13^CO_2_ in breath samples, and clearly reports sucrase activity. Further work is needed to validate it and understand its applications in evaluating EE.

## Introduction

Environmental Enteropathy (EE) is a disorder with global distribution which contributes to poor child growth, micronutrient deficiencies, and impaired responses to oral vaccines (1, 2). There is evidence that EE is associated with malabsorption of zinc, but the effects on other intestinal functions are largely unknown (3). The principal function of the intestine is to digest and absorb nutrients from the luminal stream, and several biochemical processes are essential to this dominant function. These processes include digestion of proteins into amino acids and peptides, digestion of complex carbohydrates into monosaccharides, emulsification and digestion of lipids. Nutrients, including these macronutrients together with micronutrients such as minerals and vitamins, are absorbed by epithelial cells using a very complex array of several hundred transporters. A considerable amount of research was carried out on nutrient digestion and absorption in the 1960s and 1970s, but much less has been done recently. Recent elucidation of the importance of EE in global health and nutrition has highlighted the lack of information on digestion and absorption in the world literature, and there is almost no information on the impairments of digestion and absorption related to EE in adults and children in low- and middle-income countries.

The small bowel lesion in EE has traditionally been diagnosed through histology and morphometric analysis to recognize the key features of villous remodeling with blunting, crypt hypertrophy, villous fusion, reduced villous height to crypt depth ratio, mucosal inflammation, and epithelial breaches (3, 4). However, there are clear logistical and ethical barriers to the use of endoscopy and biopsy for assessment of this lesion in asymptomatic children and adults in low-income countries, particularly for research purposes, hence surrogate markers of gut absorptive capacity and barrier function have been widely used. Dual sugar absorption tests including the lactulose: mannitol and lactulose: rhamnose tests have been used for this purpose, whereby the sugars are administered in an oral solution and recovery is measured in the urine by liquid chromatography or mass spectrometry (5). As lactulose is a large disaccharide which should not normally pass through the healthy intestinal barrier, a higher ratio of lactulose to the smaller monosaccharides mannitol or rhamnose denotes a loss of barrier function (6). This method of determining barrier function however has several limitations to widespread standardized use in low- and middle-income countries, including inconsistency of saccharides used, inconsistency of methods used to measure saccharides, and inconsistent timing of urine collection (7). Furthermore, this method provides no information about digestive capacity of the gut or about handling of sugars or amino acids or indeed other specific nutrients.

The availability of a non-invasive biomarker of functional capacity of the small intestine would be a major advantage in future research into both pathophysiology and potential interventions for EE (8). Intestinal sucrase is highly expressed in the brush border but has been shown to be impaired with villous blunting in Australian Aboriginal children, assessed by digestion of ^13^C-sucrose and recovery of ^13^C in oxidized ^13^CO_2_ in the breath and has been observed to be inversely correlated with intestinal permeability as determined by lactulose:rhamnose ratio (9). The test however has several limitations, in particular the large dose (2 g/kg up to 20 g) of naturally enriched sucrose needed to achieve a measurable signal in breath ^13^CO_2_ and the challenges of reduced signal in breath ^13^CO_2_ in regions of the world where consumption of naturally ^13^C rich foods is high. Here we sought to evaluate and validate a ^13^C-sucrose breath test (^13^C-SBT) protocol based on a small dose of highly enriched ^13^C sucrose to assess intestinal sucrase activity for the classification of physiological dysfunction in EE in a targeted population of Zambian adults.

## Materials and methods

The study was undertaken in two different settings; Glasgow, Scotland and Lusaka, Zambia. An evaluation and validation phase of the ^13^C-SBT was undertaken in healthy adult participants in Glasgow and a validation study was undertaken in healthy adults in Zambia in a community where EE is highly prevalent and where access to intestinal biopsies was ethically justifiable. This design also allowed for direct comparison of sucrose digestive capacity in a United Kingdom cohort vs. a Zambian cohort.

### Study setting in Glasgow

Two separate studies were completed in Glasgow. The first was an evaluation study comparing ^13^C-SBT kinetics when using either a 50 mg dose of highly enriched ^13^C or a larger 20 g dose of naturally enriched sucrose as outlined by Ritchie et al. (9). The second study aimed to examine the dose-response relationship of the ^13^C-SBT in combination with an intestinal sucrase inhibitor. Both studies were conducted in a healthy adult population.

### Glasgow study 1; ^13^C-sucrose breath test overview

The first study aimed to determine if the highly enriched ^13^C-SBT provided comparable results to the naturally enriched ^13^C-SBT previously reported (9) where a significant difference (*P* < 0.001) in the cumulative percentage of dose recovered was reported at 90 min (cPDR90) between Aboriginal children with diarrhea (*n* = 18) and non-Aboriginal controls (*n* = 7) using 20 g naturally enriched sucrose. A similar sized cohort (*n* = 20) was therefore recruited for the current study. As this was a pilot study, a formal sample size calculation was not possible. Participants recruited in Glasgow, United Kingdom, were aged between 18 and 65 years with no history of gastro-intestinal symptoms/disease. The study was approved by the University of Glasgow College of Medical, Veterinary and Life Sciences Research Ethics Committee (Application Number: 200170060). All Participants provided written informed consent.

### Glasgow study 1; study design and protocol

Participants completed a baseline breath test with 20 g naturally enriched sugar cane-derived sucrose (Tate and Lyle Europe, London, United Kingdom) before being randomly assigned to either the “added carrier” or “no added carrier” group. These groups completed a subsequent breath test with 50 mg highly enriched sucrose (U-^13^C sucrose, ≥ 99 atom% ^13^C, Sigma-Aldrich, Poole, United Kingdom) administered in water (200 ml) either with or without 20 g unlabeled beet sucrose (Silver Spoon, London, United Kingdom). There was a minimum 3-day washout period between each test. Participants followed a low ^13^C diet (10) for 3 days prior and were fasted for 8 h before starting each test. Two baseline breath samples were collected, participants then immediately ingested the sugar doses dissolved in water before providing breath samples every 15 min for the following 8 h. Samples were collected in 12 ml Exetainer breath-sampling vials (Labco, United Kingdom) and analyzed by isotope ratio mass spectrometry (IRMS AP-2003, Manchester United Kingdom) at the Scottish Universities Environmental Research Centre (SUERC; East Kilbride, United Kingdom) as previously described (11).

### Glasgow study 2; sucrase inhibition ^13^C-sucrose breath test overview

The second study in Glasgow aimed to further validate the ^13^C-SBT and assess its suitability when sucrase activity is inhibited, as would be expected in cases of EE. The intestinal sucrase-isomaltase (SI) inhibitor used for this study was Reducose (Phynova Group Ltd., Oxford, United Kingdom), a mulberry leaf extract standardized to contain 5% 1-Deoxynojirimycin, an active α-glucosidase inhibitory component. Mulberry leaf extracts containing 1-Deoxynojirimycin, including Reducose, have previously been shown to significantly reduce the change in peak plasma glucose following sucrose ingestion (12–15). A recent study has demonstrated a significantly (*p* < 0.001) lower peak blood glucose and peak insulin after a 75 g sucrose dose in combination with 250 mg Reducose compared to 75 g sucrose alone (16).

The second study in Glasgow recruited 18 healthy adults. This sample size was calculated using data from previous work (9) which found a mean reduction from 4.1 to 1.9% (*SD* = 2.32) in cumulative percentage of the administered dose recovered at 90 min (cPDR90). It was determined that a sample size of 18 would be required to detect a similar reduction in breath ^13^CO_2_ response with a power of 80% and α = 0.05. The study was approved by the University of Glasgow College of Medical, Veterinary and Life Sciences Research Ethics Committee (Application Number: 200190155). All Participants provided written informed consent.

### Glasgow study 2; study design and protocol

Participants completed a baseline breath test with 25 mg highly enriched sucrose (U-^13^C sucrose, ≥ 99 atom% ^13^C, Sigma Aldrich, United Kingdom) alone. They then repeated this breath test having consumed 750 mg of Reducose approximately 1 min before the 25 mg dose of ^13^C sucrose dose. There was a minimum 3-day washout period between each study day. The general protocol for each study day remained the same. Participants followed a low carbon-13 diet for 3 days prior and fasted for 8 h before starting each test. Two baseline breath samples were collected, participants then immediately ingested either the Reducose doses followed by the sugar dose, both dissolved in water, or the sugar dose alone in water before providing breath samples every 15 min for the following 8 h. Samples were collected in 12 ml Exetainer breath-sampling vials (Labco, Lampeter, United Kingdom) and analyzed by IRMS.

### Study setting in Zambia

This study was carried out from August-December 2019 in Misisi compound, an overcrowded informal settlement just south of Lusaka city center where we have conducted studies on EE since 1995. Participants were all adults who gave informed, written consent after a process which included group discussions of the study protocol and what the study involved. The study was reviewed and approved by the Excellence in Research Ethics and Science Converge Institutional Review Board (ERES Converge IRB, 2018-Dec-010).

### Zambia participant recruitment and screening

The study was advertised to one section (section B) of the community in Misisi by word of mouth, and interested adults invited to meetings to discuss the study and its procedures. All participants attended a screening interview which, alongside a physical examination, was intended to exclude potential participants with clinical illness who were then referred appropriately for medical care. Of 29 potential participants who attended for screening, 24 were recruited; others were excluded on the basis of uncontrolled hypertension (*n* = 2), cardiac murmur (*n* = 1), ovarian cyst (*n* = 1) or goiter requiring further investigation (*n* = 1).

### Zambia breath test protocol

On day 1, each participant underwent a ^13^C-SBT after an overnight fast. U-^13^C sucrose (0.3 mg/kg) was administered in water and consumed immediately at the start of the test. Breath samples were collected in duplicate at baseline, then at 15, 30, 45, 60, 90, 120, 150, 180, 210, and 240 min after ingestion ^13^C sucrose. Breath tubes were shipped to SUERC for analysis by IRMS.

### Analysis of breath test data

Curve fitting was used to develop parameters which describe the kinetics of ^13^CO_2_ evolution as previously described (11) and are by proxy an indication of the kinetics of intestinal sucrase activity as the rate-limiting step in sucrose digestion and oxidation to CO_2_. Raw breath enrichment data were combined with anthropometric data to compute ^13^C excretion rate as percentage dose recovery per hr (PDR/hr) which corrects for differences in administered ^13^C sucrose dose and scales for changes in CO_2_ production assessed by body mass. This allows direct comparison of data sets within and between study cohorts. Several previously described parameters for interpreting breath tests data (11) were used to assess ^13^C-SBT response which include the time to excretion of 50% area under the curve (AUC, T_1/2_), time to peak maximal ^13^C enrichment (T_max_), and cumulative percent dose recovered at 90 min (cPDR90) as previously described which is based on raw cumulative breath ^13^C recovery data (9).

### Endoscopy and biopsy handling

On day 2, each participant underwent upper gastrointestinal endoscopy under sedation (midazolam and pethidine), also after an overnight fast. Following routine inspection of the upper gastrointestinal tract, biopsies were taken from the second/third part of the duodenum. Three biopsies were placed in normal saline prior to orientation under a dissecting microscope and fixation in buffered 10% formal saline; two biopsies were collected into incubation medium (see below), and two were snap-frozen in liquid nitrogen. Formalin-fixed biopsies were then embedded and processed to 4 μm sections, then stained with haematoxylin and eosin for morphometry using a VS-120 scanning microscope (Olympus, Hamburg, Germany).

### Sucrase-isomaltase (A17) immunofluorescence staining

Tissue-embedded paraffin blocks to be sectioned were placed face-down on a cold plate for 20 min then sectioned on the microtome which was pre-set to cut 2–5 μm sections with the microtome blade at an angle range of 4–6°. A rough trimming of the paraffin section on the block was done before obtaining a wax ribbon with a complete section. Forceps were used to pick up the ribbon then lay it on a water bath that was pre-heated to a temperature between 35 and 37°C. This was done for a few seconds to allow the sections on the ribbon to stretch. Each section was picked onto a glass slide after being carefully separated. In order to allow the water to exit the slide and section, these sections were picked at an angle and then allowed to drain for a few minutes before being put on a hot plate to enhance adherence to the slide.

Glass slides with paraffin embedded sections were incubated at 60°C for 1 h before being taken to water, that is deparaffinized in two changes of xylene (5 min each), washed in two changes of absolute alcohol (3 min each), 70% ethanol (1 min), and distilled water (1 min).

For antigen retrieval, working dilutions were made by mixing 1 part of pH 6.0 citrate-based antigen retrieval concentrate with 9 parts of deionized/distilled water. The retrieval solution was preheated to 92–95°C. The slides were then immersed into the preheated retrieval solution and incubated for 20 min on low heat using a microwave. After incubation, the slides were washed with running tap water for 10 min after which they were permeabilized in PBS containing 0.25% Triton X at room temperature for another 10 min. The slides were further washed in PBS 4 times for 5 min each. A hydrophobic barrier was created around the tissue on the slides using a Dako pen. This step was followed by protein blocking which was done by incubating the tissue in 10% donkey serum for 30 min at room temperature. The tissues were then incubated in the primary antibody, anti-sucrase-isomaltase (A17) diluted in 10% donkey serum at a concentration of 1:50 for 2 h. This was followed by 4 washes in PBS for 5 min each. As a secondary antibody, an Alexa Fluor 488 conjugated donkey anti-goat antibody diluted in 10% donkey serum (1:50) was introduced to the tissue and incubated for 1 h then washed in PBS 4 times for 5 min each. Finally, a drop of mounting media containing DAPI was added to each tissue which was then cover slipped by a cover glass pre-cleaned with 70% ethanol. The slides were viewed using the EVOS fluorescent microscope at the magnification x20. Each slide was divided into fields then graded with a system ranging from 0 to 3, of which an average score was obtained to represent the presence of the sucrose-isomaltase (SI) enzyme.

### In vitro sucrase assay

The sucrase assay was composed of two stages adapted from a previous protocol (17). First, biopsies were placed in incubation media immediately after collection for precise periods of time, then removed and supernatants snap frozen in liquid nitrogen. Second, supernatants were transported to Glasgow where the quantity of enzymatic products was determined. The biopsy substrate media stock contained 14.5 mM sucrose (S7903; Merck, Darmstadt, Germany) dissolved in borate buffer (50 mM, pH8.5) (10628694; Fisher Scientific, Loughborough, United Kingdom) which was prepared and stored on ice shortly before use. Each 1.5 ml reaction tube contained 250 μL biopsy substrate medium stock and 250 μL Tris-HCL buffer (pH 7, 20 mM). Two biopsies were collected from each participant then placed in separate tubes and incubated at 37°C. One biopsy from each participant was incubated for 1 h, and one for 24 h. After incubation the biopsies were removed and the medium aliquoted and frozen at -80°C. One aliquot was then sent to Glasgow (United Kingdom), packaged with dry ice, for the color reaction and enzyme activity determination. To create standard curves to compare with the supernatants, known activities of invertase (0–0.25 Units) were incubated with the same substrate medium used in the biopsy study. Invertase (EC 3.2.1.26) from baker’s yeast (S. cerevisiae) (I4504; Merck, Darmstadt, Germany) was used for the standard preparations in the assay as a substitute for intestinal mucosal sucrase-isomaltase (EC 3.2.1.48) which is not readily available for purchase. Invertase is a commonly used alternative to sucrase as they both hydrolyze sucrose into fructose and glucose; invertase cleaves the O-C (fructose) bond, while sucrase cleaves the O-C (glucose) bond.

### Biopsy media color reaction and sucrase activity determination

This was undertaken in Glasgow, United Kingdom. Fifty microliter of the biopsy-substrate incubation supernatant was added to duplicate 96-well microtiter plate wells. Fifty microliter from the invertase standard incubations was also added to separate duplicate wells. Fifty microliter sucrase color reagent was then added to the microtiter wells. This color reagent was composed of fructose dehydrogenase (FDH; EC 1.1.99.11) from Gluconobacter spp. (6.65814 U/ml) (F4892; Merck, Darmstadt, Germany), thiazolyl blue tetrazolium bromide (MTT) (0.4285 mM) (M2128; Merck, Darmstadt, Germany) and phenazine methosulfate (PMS) (0.6285 mM) (10626332; Thermo Fisher Scientific, Loughborough, United Kingdom). The FDH stock was prepared in citric phosphate buffer at pH 4.5 consisting of 0.05 M citric acid (10193333; Thermo Fisher Scientific, Loughborough, United Kingdom) and 0.09 M sodium phosphate dibasic (Na_2_HPO_4_) (71640; Merck, Darmstadt, Germany) with 1% triton X-100 (11488696; Thermo Fisher Scientific, Loughborough, United Kingdom). The MTT stock was also prepared in citric phosphate buffer at pH 4.5 consisting of 0.05 M citric acid and 0.09 M sodium phosphate dibasic. The PMS stock was prepared in distilled water. Both the MTT and PMS stocks were prepared in aluminum foil wrapped vials immediately before use in the sucrase color reagent. The microtiter plate containing an invertase standard activity curve and the biopsy supernatants was then incubated in the dark at 37°C for 40 min before the absorbance at 570 nm was measured (MultiSkan Spectrum V1.2, Thermo Fisher Scientific). The spectrophotometer was programmed to shake the plate at a moderate rate for 3 s before the absorbance reading.

### Testing for proximal small intestinal fermentation of sucrose

To search for evidence of proximal small intestinal sucrose fermentation, 19 Zambian adults underwent a hydrogen breath test with 50 g sucrose, using a Gastrolyzer handheld monitor (Bedfont Scientific, United Kingdom) monitored over 4 h.

### Data analysis

Results were analyzed using Statistical Package for Social Sciences (SPSS) version 25 (IBM, New York, United States). Graphs were produced on Microsoft Excel 365. Data were first assessed for normality by the Kolmogorov-Smirnov test.

Enzyme activities of the supernatants were calculated using Microsoft Excel. Standard curve absorbances were plotted against known enzyme activities. The absorbances of the samples with unknown activities could then be interpolated based on linear regression modeling of the standard curve using the following equation;


I⁢n⁢t⁢e⁢r⁢p⁢o⁢l⁢a⁢t⁢e⁢d⁢e⁢n⁢z⁢y⁢m⁢e⁢a⁢c⁢t⁢i⁢v⁢i⁢t⁢y=

$$\frac{\left(S\,a\,m\,p\,l\,e\,a\,b\,s\,o\,r\,b\,a\,n\,c\,e-S\,t\,a\,n\,d\,a\,r\,d\,c\,u\,r\,v\,e\,Y\,i\,n\,t\,e\,r\,c\,e\,p\,t\right)}{S\,t\,a\,n\,d\,a\,r\,d\,c\,u\,r\,v\,e\,g\,r\,a\,d\,i\,e\,n\,t}$$

Anomalies outside the standard curve range were removed from analysis. Correlations between biopsy enzyme activities, biopsy morphometry and Zambian participant breath parameters were determined using the Pearson correlation coefficient. Analysis of the 24-h biopsy assay incubation were excluded due to instability of the assay incubation constituents over the 24-h period (inter-assay coefficients of variation 30.81 vs. 13.28%).

Baseline characteristics of the added carrier and no added carrier group participants from study 1 in Glasgow were compared with one-way ANOVA. Differences in breath parameters between the 50 mg highly enriched and 20 g naturally enriched sucrose doses within the “added carrier” and “no added carrier” groups were assessed using one-way ANOVA with repeated measures. The differences in breath parameters between the “added carrier” and “no added carrier” group were compared using one-way ANOVA.

Breath parameters from Study 2 in Glasgow (^13^C-SBT with Reducose^®^) were compared using one-way ANOVA with repeated measures.

## Results

### Glasgow results

#### Study 1-participant details

Nineteen participants completed study 1 in Glasgow (10 M, 9 F). Their anthropometric characteristics are outlined in Table 1. There were no significant differences (*P* > 0.05) in height, weight, body mass index (BMI), age or gender between the “Added carrier” and “No added carrier” groups.

**TABLE 1:** Baseline participant characteristics for Glasgow participants (study 1).

Characteristic, mean (*SD*)	No added carrier group (*N* = 11)	Added carrier group (*N* = 8)	*P*-value
Height (cm)	173.76 (8.15)	168.71 (7.3)	0.182
Weight (kg)	69.1 (8.53)	60.86 (19.87)	0.233
BMI, mean (kg/m^2^)	22.85 (1.88)	21.09 (5.15)	0.310
Age, mean (y)	22.91 (4.32)	22.75 (4.86)	0.941
Male: female	7:4	2:6	0.106

#### Highly enriched vs. naturally enriched sucrose breath test results

**FIGURE 1:**
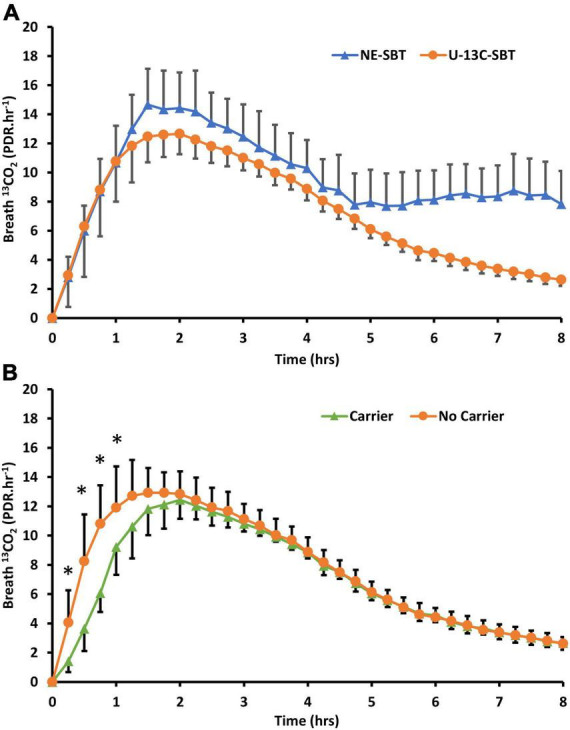
**(A)** Breath ^13^CO_2_ evolution (PDR.Hr^–1^) from participants (n = 19) who consumed either 20g of naturally enriched sucrose (NE-SBT) or 50mg of U-^13^C-sucrose (U^13^C-SBT) in a randomized cross over design. **(B)** Of the 20 participants consuming 50mg U-^13^C-sucrose some consumed 50mg U-^13^C-sucrose alone (No Carrier, n = 11) or 50mg U-^13^C-sucrose with 20g of beet sucrose (Carrier, n = 8) to compare the effect of additional sucrose on breath ^13^CO_2_ response, * denotes a significant difference between groups (P < 0.05).

The preliminary study in Glasgow showed a highly enriched 50 mg dose of fully labeled ^13^C sucrose (U-^13^C sucrose) provided comparable breath results to a 20 g naturally enriched sucrose dose (Figure 1A). There were no significant differences (*P* > 0.05) in the time to 50% dose recovery (T_1/2_), mean area under the curve (AUC) or cumulative percentage dose recovered at 90 min (cPDR90) between these two sugar doses alone (Table 2). The time of maximum breath enrichment (T_*MAX*_) was significantly greater after consuming 20 g naturally enriched sucrose compared to the 50 mg U-^13^C sucrose alone. Additionally, when the 50 mg dose of U-^13^C sucrose was consumed alongside 20 g unlabeled sucrose (added carrier, Figure 1B), the AUC and cPDR90 were significantly lower than the 20 g naturally enriched sucrose dose (*p* < 0.05) (Table 2). The 50 mg dose of U-^13^C sucrose yielded approximately ∼5.6 × greater breath enrichment (span from baseline to peak enrichment) compared to the 20 g naturally enriched sucrose dose. When comparing the U-^13^C sucrose breath results between the added carrier and no added carrier group, the additional unlabeled carrier resulted in a significantly lower AUC and cPDR90 compared to U-^13^C sucrose with no added carrier (*P* < 0.05) (Table 2). Consuming the additional unlabeled carrier alongside the U-^13^C sucrose also resulted in a significantly greater T_*MAX*_ (<0.05). Together the breath enrichment and comparable breath results of the 50 mg highly enriched fully labeled sucrose tracer highlight its suitability as an alternative to the 20 g naturally enriched sucrose.

**TABLE 2:** Summary breath parameters from participants (*N* = 19) who consumed 20 g naturally enriched sucrose (NES) or 50 mg highly enriched sucrose (HES) either with an added carrier dose or without an added carrier dose.

	No added carrier group (*N* = 11)			Added carrier group (*N* = 8)			
		
	20 g NES mean (*SD*)	50 mg HES mean (*SD*)	*P*-value	20 g NES mean (*SD*)	50 mg HES mean (*SD*)	*P*-value	50 mg HES, carrier vs. no carrier *P*-value
AUC	66.72 (17.93)	61.56 (4.37)	0.35	67.25 (8.36)	55.95 (2.95)	0.01	0.01
CPDR90	12.33 (2.93)	13.57 (3.13)	0.31	12.08 (2.14)	9.22 (1.54)	<0.01	<0.01
T_*MAX*_	1.97 (0.26)	1.62 (0.35)	0.03	1.97 (0.22)	2.05 (0.29)	0.17	0.01
T_1/2_	3.35 (0.71)	3.21 (0.34)	0.52	3.56 (0.60)	3.38 (0.22)	0.40	0.23

### Sucrase-isomaltase (Reducose) inhibitor study

#### Study 2 – participant details

Eighteen participants (9 M, 9 F) completed study 2 in Glasgow. Their anthropometric characteristics are outlined in Table 3.

**TABLE 3:** Baseline participant characteristics for Glasgow participants (study 2).

Characteristic	Mean (*N* = 18)	*SD*
Height (cm)	169.7	8.7
Weight (kg)	71.0	15.6
BMI, mean (kg/m^2^)	24.6	5.0
Age, mean (y)	24.8	5.1
Male: female	9:9	

#### Sucrase inhibitor study breath test results

**FIGURE 2:**
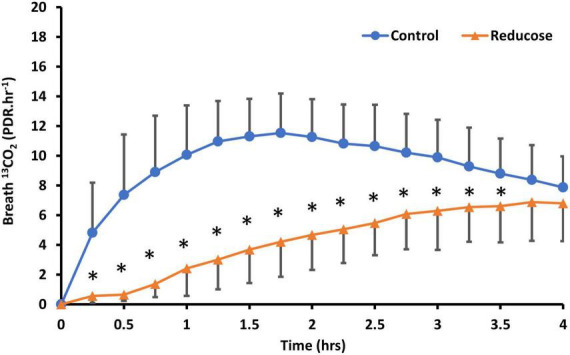
Comparison of U-^13^C-sucrose breath ^13^CO_2_ response (PDR.HR^–1^) in participants (N = 18) ingesting 50 mg U-^13^C-sucrose or 50mg U-^13^C-sucrose with Reducose (750 mg) taken approximately 1 min before ingestion of the tracer. Results are presented as mean (SD), * denotes significant differences between control and Reducose (P < 0.05).

Consuming 750 mg of Reducose significantly reduced both the rate and extend of ^13^CO_2_ production from sucrose digestion (Figure 2). The impact was clearly reflected in the kinetic parameters derived from the breath test curves (Table 4).

**TABLE 4:** Summary breath parameters from participants (n = 18) who consumed placebo and Reducose (750 mg) in a single-blinded, randomized cross-over study.

	Control (N = 18)		Reducose (N = 17)		
		
	Mean	SD	Mean	SD	p
T_max_	1.55	0.55	3.22	1.20	<0.01
T_1/2_	3.38	0.73	4.75	2.01	0.01
4 h AUC	36.96	8.36	16.22	5.77	<0.01
cPDR90	11.94	4.06	2.49	1.46	<0.01

### Zambia results

#### Participants

Participating adults included 11 men and 13 women (Table 5) aged between 18 and 59 years. Nutritional status varied widely: body mass index (BMI) ranged from 17.9 to 49.3 kg/m^2^.

**TABLE 5:** Basic characteristics of study participants in Lusaka.

	Men (n = 11)	Women (n = 13)	P
Age (years)	34.81 (15.94)	44.08 (10.48)	0.10
BMI (kg/m^2^)	20.53 (1.90)	30.17 (7.9)	<0.01
VH (μ m)	231.19 (44.19) (n = 10)	219.24 (32.57) (n = 12)	0.47
CD (μ m)	163.15 (34.47) (n = 10)	143.02 (37.64) (n = 12)	0.21
ESA (μ m)	605.43 (117.15) (n = 10)	588.42 (91.40) (n = 12)	0.71
Sucrase expression observed by immunofluorescence	8	8	0.68

Values shown are mean (SD).

#### Breath excretion of ^13^CO_2_

**FIGURE 3:**
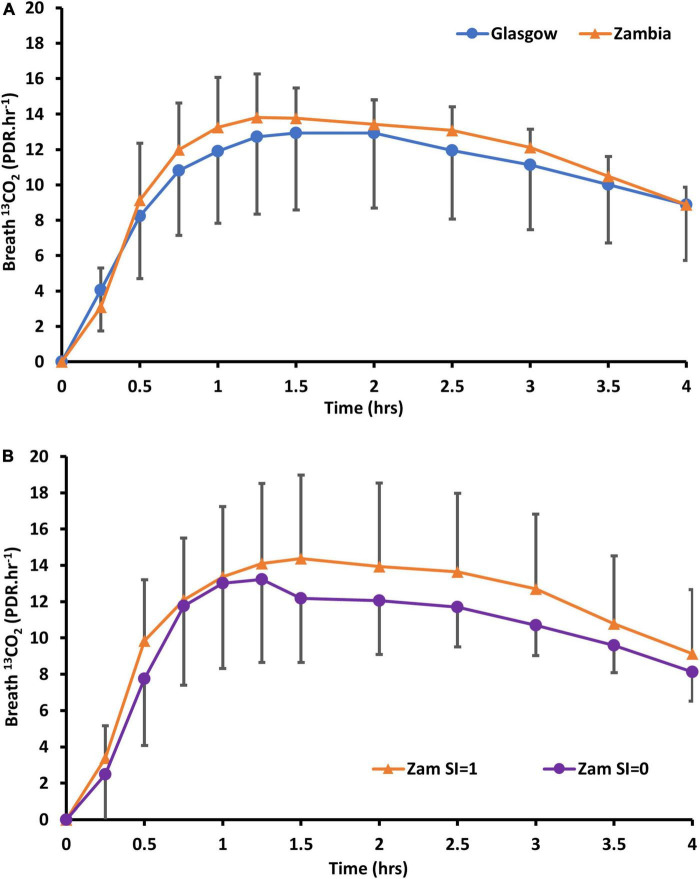
Delta over baseline curves of ^13^CO_2_ in **(A)** Zambian adults following oral dosing with ^13^C_12_-sucrose, compared to healthy Glasgow participants (without carrier) and **(B)** Zambian participants alone whose biopsies did (SI = 1) or did not (SI = 0) show sucrose-isomaltase immunostaining.

Sample collection was complete in all cases, with ^13^C excretion in breath showing the anticipated pattern (Figure 3).

#### Mucosal morphometry

**FIGURE 4:**
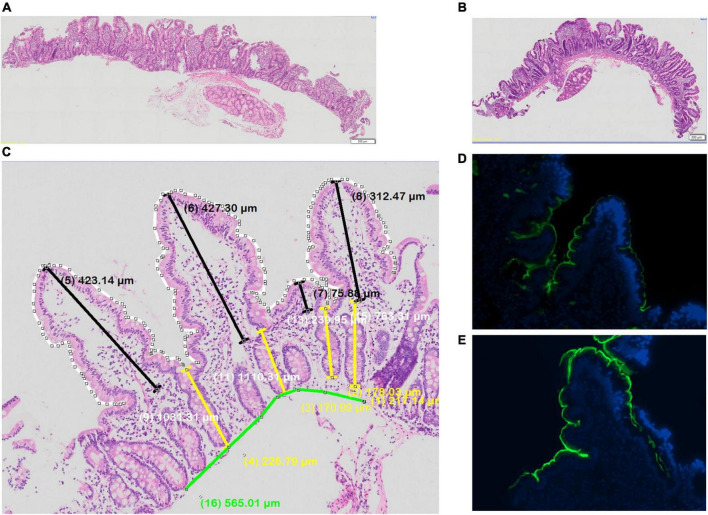
Biopsies from adult Zambian study participants **(A,B)** histology, showing variable villus blunting; **(C)** morphometry; and **(D,E)** immunostaining for sucrose-isomaltase, showing lower **(D)** and higher **(E)** protein expression.

Biopsies showed villus blunting, crypt lengthening, lamina propria inflammation and epithelial abnormalities, consistent with previous work in this community (Figure 4). Morphometry was satisfactory in 18 (75%) of cases, as some biopsies were too small or poorly orientated to permit formal morphometry.

#### Breath test performance against sucrase-isomaltase immunostaining

**FIGURE 5:**
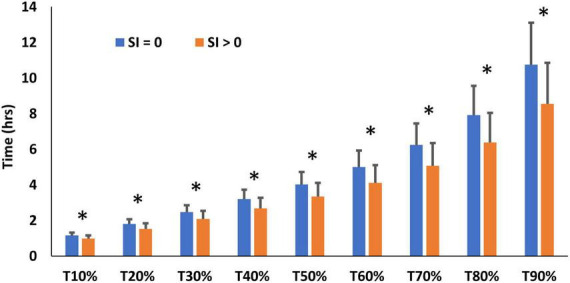
SBT performance (time to recovery of x% of tracer) in participants whose biopsies did (*SI* > 0) or did not (*SI* = 0) show sucrose-isomaltase immunostaining, * denotes significant differences between *SI* > 0 and *SI* = 0 (*P* < 0.05).

Immunostaining for SI was obtained in one biopsy in all 24 cases, but for a second biopsy in only 20 cases. Immunostaining (Figure 4) was seen in the majority of biopsies (Table 4). Time to recovery of a given fraction of the total dose administered was shorter in the participants whose biopsies showed SI immunostaining (Figure 5).

#### Breath test performance against biopsy morphometry

No significant correlations were found between the biopsy morphometries and breath test parameters(Table 6).

**TABLE 6:** Pearson correlations between participant’s biopsy morphometry and breath test parameters.

	Unadjusted correlation (*r*)	*P*	Adjusted correlation (*r*)*^a^*	*P*
VH vs. cPDR90	0.258	0.353	0.166	0.607
VH vs. T_1/2_	-0.322	0.208	-0.149	0.612
CD vs. cPDR90	0.341	0.213	0.420	0.740
CD vs. T_1/2_	-0.280	0.277	-0.259	0.372
VH/CD vs. cPDR90	-0.152	0.589	-0.303	0.338
VH/CD vs. T_1/2_	0.126	0.630	0.246	0.397
ESA vs. cPDR90	-0.064	0.190	0.202	0.528
ESA vs. T_1/2_	-0.114	0.662	-0.311	0.278

^a^Adjusted for Age, Sex and BMI. VH, Villous height; CD, Crypt depth; ESA, epithelial surface area; cPDR90, Cumulative percentage dose recovered at 90 min; T_1/2,_ time to 50% dose recovery.

#### Sucrase activity against biopsy morphometry

There was a positive correlation (*r* = 0.565, *P* = 0.070) between biopsy sucrase activities and villus height which was significant when adjusted for the participants age, sex and body mass index (BMI) (*r* = 0.724, *P* < 0.05), which appeared to be driven primarily by BMI and age. Additionally, there was a significant unadjusted correlation between sucrase activity and epithelial surface area (ESA) (*r* = 0.702, *P* < 0.05) (Table 7).

**TABLE 7:** Pearson correlations between participant’s biopsy morphometries and sucrase activities.

	Unadjusted correlation (*r*)	*P*	Adjusted correlation (*r*)*^a^*	*P*
SA vs. VH	0.565	0.070	0.724	0.042*
SA vs. CD	-0.041	0.904	-0.255	0.542
SA vs. VH/CD	0.455	0.159	0.701	0.053
SA vs. ESA	0.702	0.016*	0.584	0.128

^a^Adjusted for Age, Sex and Body Mass Index (BMI). SA, Sucrase activity [Units/biopsy wet weight (mg)]; VH, Villous height; CD, Crypt depth; ESA, epithelial surface area. *Significant correlation (P < 0.05).

#### Sucrase activity against breath parameters

No statistically significant correlations were found between biopsy sucrase activities and breath parameters (*P* > 0.05) (Table 8).

**TABLE 8:** Pearson correlations between biopsy sucrase activities and breath test parameters.

	Unadjusted correlation (*r*)	*P*	Adjusted correlation (*r*)*^a^*	*P*
SA vs. cPDR90	-0.455	0.160	-0.285	0.494
SA vs. T_1/2_	0.158	0.624	-0.069	0.859

^a^Adjusted for Age, Sex and Body Mass Index (BMI). SA, Sucrase activity [Units/biopsy wet weight (mg)]; cPDR90, Cumulative percentage dose recovered at 90 min; T_1/2,_ time to 50% dose recovery.

#### Testing for proximal small intestinal fermentation of sucrose

Once it was apparent that the ^13^C-SBT curves were very similar in British and Zambian adults it was decided to search for evidence of fermentation of sucrose which could explain an early peak in ^13^CO_2_ or confound interpretation of the ^13^C-SBT. Of 14 sucrose breath tests carried out, 7 had high (<20 ppm) baseline readings, before the sucrose was administered, and only one showed a rise of > 20 ppm increment from baseline consistent with fermentation of the ingested sucrose.

## Discussion

EE remains an elusive sub-clinical pathophysiological condition affecting the gut in populations throughout low-and-middle-income countries (LMICs). Beyond visual and histological analysis of the gut architecture, which is only accessible in limited research settings, testing for EE in communities remains challenging and therefore the role of EE in malnutrition remains unclear. Here we sought to develop a new non-invasive stable isotope based breath test using ^13^C sucrose to assess the degree of epithelial damage, a hallmark of EE (18). The results demonstrate that a very small dose of highly enriched sucrose is sufficient to measure intestinal brush-border enzyme activity *in vivo*. This has advantages in studies in young children, where EE plays a prominent role in growth failure (19) and administration of a small dose of sucrose (a few milligrams) is more feasible than using a large dose of naturally enriched sucrose. The ^13^C-SBT faithfully detects inhibition of sucrase-isomaltase activity. This is also supported by the observation of reduced ^13^CO_2_ response in the ^13^C-SBT test in congenital sucrase-isomaltase deficiency (20). Applying the ^13^C-SBT in a community setting with high EE prevalence where access to concomitant biopsy was ethically justifiable revealed that ^13^C-SBT was able to distinguish between gross sucrase-isomaltase deficiency and competent brush-border enzyme activity, as detected by immunostaining. However, in this pilot adult cohort, the relationship between measured enzyme activity in the gut and ^13^C-SBT parameters were weak and the comparison between ^13^C-SBT in Glasgow adults and Lusaka adults suggest that the ability to digest and oxidize sucrose is maintained even when EE prevalence is high in the community.

There is a need for a routine, preferably non-invasive, test to assess small intestinal function that is suitable for large scale field work to understand how EE impacts community health. ^13^C-SBT measures two processes—brush border sucrose hydrolysis by SI and glucose/fructose absorption through SGLT1 (SLC5A1) and GLUT5 (SLC2A5). Nutrient digestion and absorption are important domains of small intestinal function and direct measurement may help in quantifying functionally important malabsorption. SI is an enterocyte-specific, brush-border enzyme that is maximally expressed as enterocytes mature and migrate to the top of the villus (21) and the villus blunting and microvillus damage observed in EE would suggest that SI is a good potential diagnostic target (18, 22). Previous work in Zambian children has also demonstrated that gene expression of sucrase-isomaltase is lower in stunted children (and markedly lower in severe acute malnutrition) compared with adults and expression of glucose transport enzymes (SLC5A1) appears to be upregulated in EE (23). Thus ^13^C-SBT may more accurately reflect SI activity in the brush-border. This was borne out in our comparison of immunofluorescent staining of biopsies for SI activity and ^13^C-SBT output. However, the correlation with measured brush border enzyme activity was neither significant nor in the expected direction. This may reflect a number of pathophysiological features of EE. Pinch biopsies were sampled from areas of the proximal small intestine where EE is thought to manifest more prominently (18). ^13^C sucrose emptying in a liquid stream from the stomach will rapidly traverse the proximal and mid small intestine and thus regions of the gut where a competent brush border would be expected to rapidly hydrolyze ^13^C sucrose. Thus, the ability of the test to capture the localized proximal damage to the gut may be limited to severe cases of EE. The small intestine in EE is also thought to harbor greater microbial diversity and capacity to metabolize nutrients and thus bacterial metabolism of sucrose by the gut microbiota would also yield ^13^CO_2_ and could be a confounding factor affecting test sensitivity (24). Small intestinal fermentation of sucrose would also produce H_2_, however, we found very limited evidence of this. Thus, a 4-h time frame is proposed for the ^13^C-SBT to capture mainly small intestinal events.

An interesting observation in our application of the large dose natural abundance sucrose dose (Figure 1A) suggests that the later rise observed in breath ^13^CO_2_ may be related to colonic fermentation of part of the sucrose load; the timing of this later peak is consistent with oro-caecal transit time (25). This effect was not observed in the small dose of highly enriched ^13^C sucrose even in the presence of the 20 g unlabeled carrier sucrose. The effect of the carrier sucrose was however observed in the early stages of breath ^13^CO_2_ production. This may be explained by delayed gastric emptying where sucrose has been shown to have a dose-dependent effect of slowing gastric emptying through the pyloric sphincter (26). A further argument in favor of a minimum dose of sucrose to sample sucrase-isomaltase activity specifically in the proximal small intestine is the observation that EE appears to cause maximal epithelial damage in the proximal small intestine (18) thus limiting sampling to the most proximal region of the small intestine may improve test performance.

Brush border enzymes remain attractive targets for measurement of abnormalities in small intestinal digestion. The surface area of the intestine is hugely magnified by three levels of amplification: valvulae conniventes (folds visible to the naked eye), villi, and microvilli. In EE the villus surface area is reduced by villus blunting and fusion, and the microvilli are damaged (22). Consequently, we anticipated being able to detect this reduced surface area in Zambian adults from a community which we have shown in previous studies to have near-ubiquitous enteropathy (3, 27, 28) compared to British adults. We did not find a clear-cut reduction in sucrase activity measured by ^13^C-SBT between Glasgow and Lusaka in any of the parameters measured. This may signify that sucrase activity is expressed at a very high level and is still satisfactory even in the presence of enteropathy, or that activity in the distal gut is enough to compensate for reductions more proximally. It is also possible that villus morphology, immunostaining, and enzyme activity measurements in pinch biopsies do not reflect the overall enzymatic capacity of the intestine with sufficient precision. However, the lack of discrimination between UK and Zambia suggest that the latter explanation is insufficient.

## Data availability statement

The raw data supporting the conclusions of this article will be made available by the authors, without undue reservation, by contacting the corresponding author(s).

## Ethics statement

The studies involving human participants were reviewed and approved by the University of Glasgow College of Medical, Veterinary and Life Sciences Research Ethics Committee and the Excellence in Research Ethics and Science Converge Institutional Review Board (ERES Converge IRB). The patients/participants provided their written informed consent to participate in this study.

## Author contributions

VO, PK, and DM involved in designing an IAEA Coordinated Research Programme (CRPE41016) which in part funded this research as part of a larger study on EE. RS, CE, and DM designed and conducted the studies in Glasgow and analyzed the data from the Glasgow studies. RS analyzed biopsy supernatants from Zambia. SM, CM, MT, PH, EB, KC, and PK involved in designing and conducting the study in Zambia. RS, PK, and DM involved in analyzing and interpreting the ^13^CO_2_ breath data in relation to the Zambia data. All authors contributed to writing the manuscript and agreed the final version.
